# Diphenylpyraline Derivatives Are Allosteric Agonists of the CB_1_ Cannabinoid Receptor

**DOI:** 10.3390/molecules31203699

**Published:** 2026-10-15

**Authors:** Khalil Eldeeb, Jill K. Harp, Ryan W. Fitzgerald, Sandra Leone-Kabler, Tony E. Reeves, Sara R. Jones, Steve R. Childers, Allyn C. Howlett, Victor M. Pulgar

**Affiliations:** 1 Jerry M. Wallace School of Osteopathic Medicine, Campbell University, Lillington, NC 27506, USA; keldeeb@methodist.edu; 2 Department of Physiology & Pharmacology (Translational Neuroscience), Wake Forest University School of Medicine, Winston-Salem, NC 27157, USA; sandra.leonekabler@advocatehealth.org (S.L.-K.); sara.jones@wfusm.edu (S.R.J.); srchilders928@gmail.com (S.R.C.); allyn.howlett@advocatehealth.org (A.C.H.); 3 Faculty of Medicine, Al-Azhar University, New Damietta 34518, Egypt; 4 Biomedical Research Infrastructure Center, Winston-Salem State University, Winston-Salem, NC 27101, USA; harpj@wssu.edu (J.K.H.); fitzgeraldrw@wssu.edu (R.W.F.); 5 Department of Biological Sciences, Winston-Salem State University, Winston-Salem, NC 27101, USA; 6 Department of Chemistry, Winston-Salem State University, Winston-Salem, NC 27101, USA; 7 Wake Forest Institute for Regenerative Medicine, Wake Forest University School of Medicine, Winston-Salem, NC 27157, USA; tony.reeves@advocatehealth.org; 8 Department of Obstetrics and Gynecology, Wake Forest University School of Medicine, Winston-Salem, NC 27157, USA; 9 Department of Pharmaceutical & Clinical Sciences, Campbell University, Lillington, NC 27506, USA

**Keywords:** adenylyl cyclase, biased agonists, cholesterol consensus motif (CCM), diphenylmethoxypiperidine, diphenylpyraline (DPP), extracellular signal-regulated kinase (ERK), G protein-coupled receptors (GPCR), mesenteric resistance arteries, pregnenolone, SR141716, WIN55212-2

## Abstract

Diphenylpyraline analogs JKH1 and JKH10 are monoamine transporter inhibitors and promote relaxation in mouse mesenteric arteries. Here, we evaluated JKH1 and JKH for their interaction with CB_1_ cannabinoid receptor signaling. In N18TG2 mouse neuroblastoma cells that endogenously express CB_1_ receptors, JKH1 and JKH10 stimulated [^35^S]GTPγS binding in a concentration-dependent manner; this stimulation was non-competitively antagonized by the CB_1_ antagonist-inverse agonist SR141716 (1 µM). JKH10 (but not JKH1) inhibited forskolin-stimulated cAMP accumulation with efficacy as high as the full orthosteric agonist WIN55212-2, and this Gi-mediated inhibition was completely precluded by pertussis toxin treatment. However, JKH1 was a poor inhibitor. The SR141716 failed to completely block the inhibition by JKH10. Both JKH analogs stimulated ERK phosphorylation, but inhibition by SR141716 (1 µM) did not appear to be overcome by increasing concentrations of JKH1 or JKH10. In mouse isolated mesenteric arteries, both analogs (1 µM) attenuated the contractile response, but only the response to JKH10 was blocked by the CB_1_ antagonist-inverse agonist O-2050 (1 µM). Docking studies to protein data bank CB_1_ structures 7FEE and 6KQI showed JKH1 was predicted to dock predominantly at a pregnenolone-binding site, whereas JKH10 was predicted to dock at both the pregnenolone and a cholesterol consensus motif at helices 2–3–4 inner membrane leaflet. Thus, a series of diphenylpyraline derivatives can behave as CB_1_ receptor stimulators, and *in silico* evidence supports that this could result from interaction with cholesterol and/or pregnenolone protein surface binding sites rather than the orthosteric binding site.

## 1. Introduction

Rational drug design was employed using the structural features of tropane cocaine to investigate pharmacotherapy to overcome drug abuse disorder. Hence, a small-molecule approach led to the initial study of diphenylpyraline (DPP), which exhibited locomotor stimulation effects like psychostimulants and was shown to act as a dopamine transporter reuptake inhibitor, increasing nucleus accumbens dopamine in vivo [1]. Studies of DPP analogs were undertaken for structure–activity relationship studies, which identified analogs that, compared with DPP, showed increased locomotor activity in mice and greater binding to the dopamine transporter in rat striatal membranes [2]. Binding of these analogs to the serotonin transporter was approximately 20-fold less potent in these determinations (Table S1).

Select DPP analogs were also examined in an ex vivo model of mouse mesenteric resistance arteries and found to inhibit adrenergic or KCl-evoked vasoconstriction [3]. The most efficacious analogs were 4-[(4-chlorophenyl, phenyl) methoxy]-1-methylpiperidine (JKH1) and 4-[bis(4-chlorophenyl)methoxy]-1-butylpiperidine (JKH10). Our group also found that vasorelaxation in the mesenteric resistance arteries can be similarly evoked by the CB_1_ cannabinoid agonists Δ9-tetrahydrocannabinol and aminoalkylindole JWH-073 [4]. Thus, it became apparent that CB_1_ receptor cellular signaling might have a role in mediating the diphenylpyraline analog responses at the molecular level.

The CB_1_ cannabinoid receptor is the most abundant G protein-coupled receptor (GPCR) in the brain and modulates processes in neurodevelopment and synaptic neurotransmission [5,6]. Although cannabinoid agonists provide benefits for pain relief, their use is limited by untoward side effects of sedation, muddled thinking, memory disruption and the potential to exacerbate mental illnesses [7,8].

**Figure 1:**
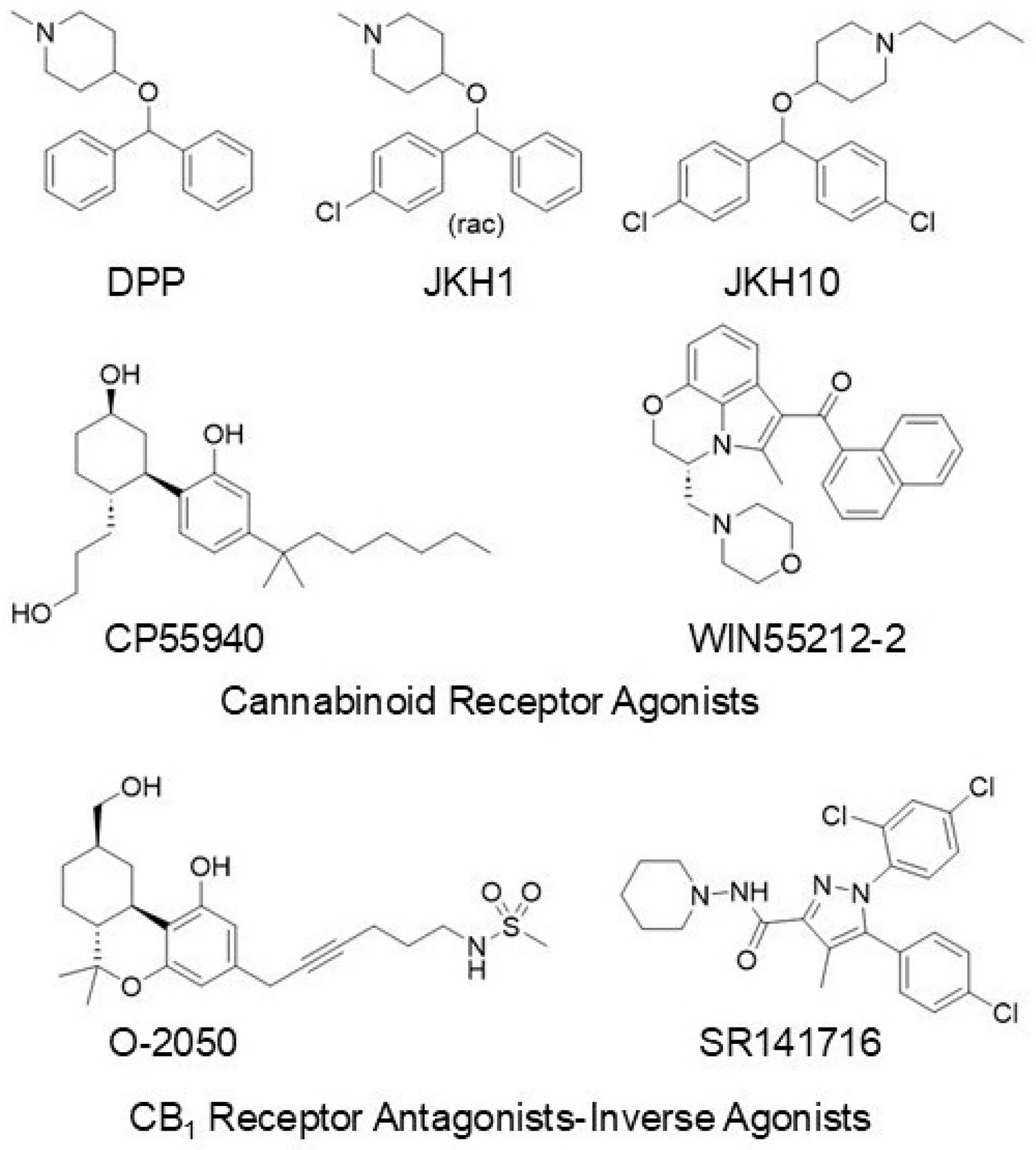
Derivatives of DPP and CB_1_ orthosteric agonists and antagonists. Several pharmacological tools are available for studying the endocannabinoid system. CP55940 is a CB_1_ and CB_2_ cannabinoid receptor full agonist that reproduces THC-like effects. WIN55212-2, despite its aminoalkylindole structure, is also an efficacious CB_1_ and CB_2_ cannabinoid agonist. SR141716 (rimonabant) and O-2050 both act as CB_1_ receptor competitive antagonists and inverse agonists [5].

The present investigation explores two DPP analogs, JKH1 and JKH10 (Figure 1), on CB_1_-mediated signal transduction in N18TG2 cultured neuronal cells that endogenously express CB_1_ receptors as well as enzymes that conduct signaling pathways via Gi proteins. At the outset, it was entirely unclear how JHK compounds that behave as monoamine transporter inhibitors might also function via the CB_1_ receptor signaling in other aspects of the cell such as the CB_1_ agonist role in vasorelaxation. However, unbiased docking studies identified that these diphenylpyraline analogs can engage pregnenolone- and cholesterol-binding sites along the outer surfaces of the CB_1_ receptor at the inner leaflet of the plasma membrane. We propose that interaction at these non-orthosteric binding site(s) may be capable of evoking stimulation or biased stimulation of the CB_1_ receptor signaling in addition to other transmembrane proteins including monoamine reuptake transporters.

## 2. Results

### 2.1. Effects of JKH1 and JKH10 on Total G Protein Activity

**Figure 2:**
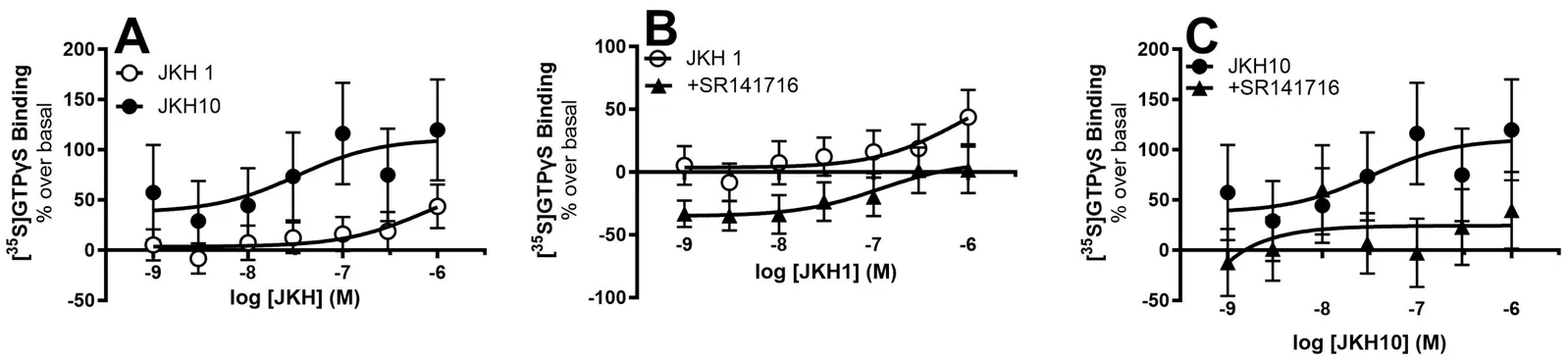
Effects of JKH1 and JKH10 on [^35^S]GTPγS binding in N18TG2 cell membranes. [^35^S]GTPγS assays to quantify ligand-mediated coupling to total G proteins were performed in N18TG2 cell membranes in the presence of varying concentrations of JKH1 or JKH10 (**A**). Effects of the CB_1_ antagonist–inverse agonist SR141716 (SR, 1 μM) on JKH1 (**B**) and JKH10 (**C**) are shown. Nonspecific binding measured in the presence of 10 μM GTPγS was subtracted from total binding to obtain specific binding. Ligand-stimulated values were transformed to “% over basal” [% = ((stimulated/basal) × 100) − 100)]. Data are shown as the mean ± SEM values of three or more independent experiments.

To determine the effects of diphenylpyraline derivatives on G protein activation, the first step in signaling from the CB_1_ receptor, [^35^S]GTPγS binding to Gα proteins was examined in N18TG2 neuronal membranes. JKH10 stimulated [^35^S]GTPγS binding in a concentration-dependent manner with an EC_50_ = 33 nM, but JKH1 exhibited poor efficacy or potency (EC_50_ > 1 μM) (Figure 2A). The JKH10 stimulation was non-competitively reduced by co-incubation with SR141716 (1 μM), as would be expected for the inverse agonist activity of SR141716 at the CB_1_ receptor (Figure 2B,C). No shift in the log dose-response curve for JKH10 was observed, indicating that SR141716 did not behave as a competitive antagonist at the binding site for JKH10. Because SR141716 competes for binding of CB_1_ orthosteric agonists, one can infer that JKH10 does not bind to the CB_1_ orthosteric binding site.

### 2.2. Effects of JKH1 and JKH10 on Forskolin (FSK)-Stimulated cAMP Accumulation

**Figure 3:**
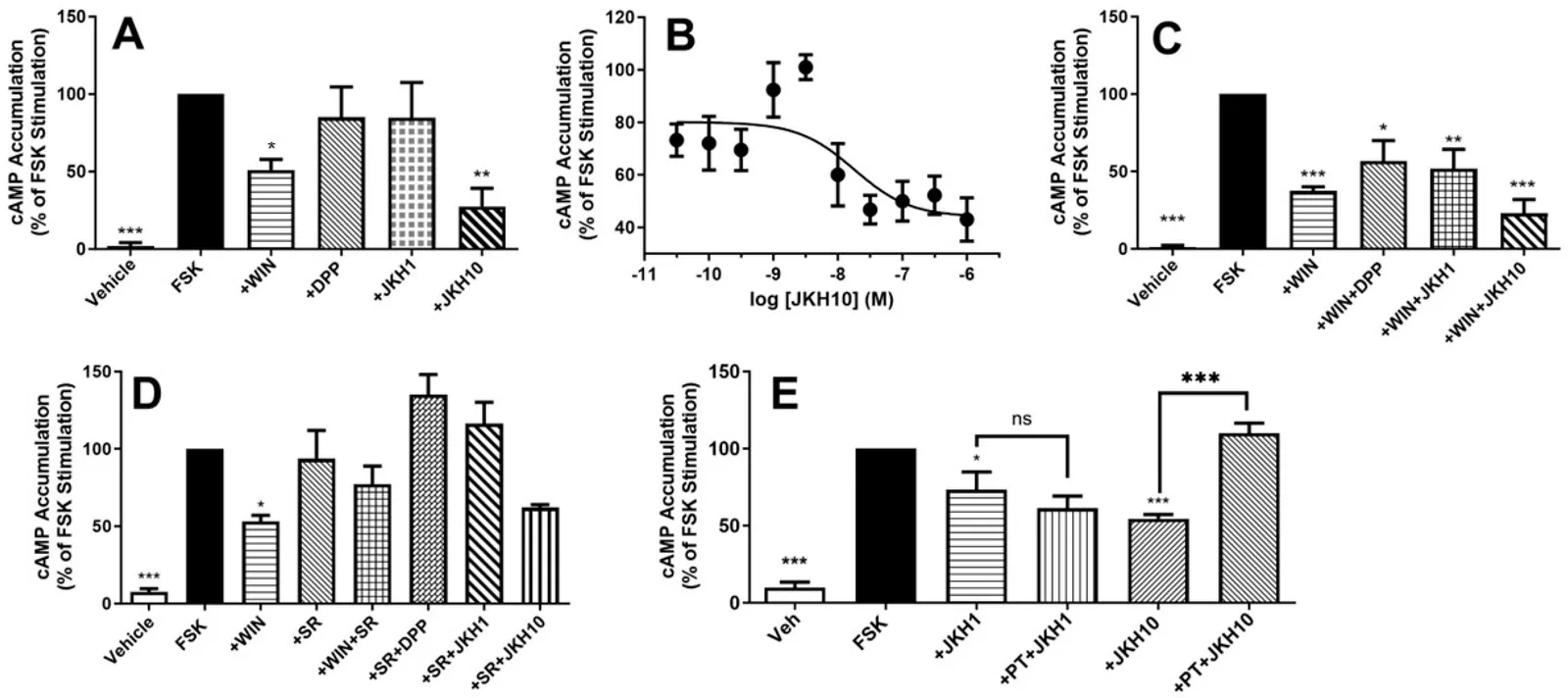
Effects of JKH1 and JKH10 on FSK-stimulated cAMP accumulation. (**A**) cAMP levels were determined by incubating N18TG2 cells with vehicle or FSK 1 μM for 4 min and in the presence of WIN55212-2 (WIN, 100 nM), DPP, JKH1, or JKH10 (100 nM); (**B**) concentration response curve to JKH10 (0.03 nM to 1 μM); (**C**) effects of JKH1 and JKH10 on WIN55212-2 (100 nM) responses; (**D**) effects of the CB_1_ antagonist SR141716 (1 µM); (**E**) effects of pre-treatment with pertussis toxin (100 ng/mL for 18 h) on JKH1 and JKH10 responses. Data were normalized such that 100% was equal to cAMP in WT cells treated with FSK only. * *p* < 0.05, ** *p* < 0.01, *** *p* < 0.001 indicate a significant difference between FSK-only treated cells and cells treated with either vehicle alone or cells treated with indicated ligands in the presence of FSK. Not significant difference is denoted as ns. Data are shown as the mean ± SEM values of three or more independent experiments.

CB_1_ receptor agonists regulate adenylyl cyclase via Gi-mediated inhibition to reduce cellular cAMP. We tested the diphenylpyraline derivatives for their ability to affect cAMP accumulation in neuronal cells. While CB_1_ full agonist WIN55212-2 (100 nM) inhibited FSK-induced cAMP accumulation (to 51 ± 6.9%), DPP or JKH1 (100 nM) were not statistically reduced (to 85 ± 19% and 84 ± 22%, respectively) (Figure 3A). On the other hand, JKH10 (100 nM) inhibited cAMP accumulation significantly (to 27 ± 11%), with an EC_50_ = 13 nM (Figure 3B). The co-incubation of maximally effective concentrations of JKH10 (100 nM) with WIN55212-2 (100 nM) augmented the WIN55212-2 response, but this difference was not statistically significant (Figure 3C). This experiment does not support activity that can be described as additive or synergistic. The poorly efficacious JKH1 failed to reverse the inhibition of cAMP accumulation by the maximally efficacious WIN55212-2, indicating that JKH1 did not behave as a competitive antagonist of the WIN55212-2 response.

SR141716 (1 µM) reversed the inhibition of cAMP accumulation in response to WIN55212-2, as expected for a competitive antagonist. As a control, SR141716 did not affect FSK-stimulated cAMP accumulation (93 ± 18%) in the absence of the orthosteric agonist. SR141716 did not sufficiently block the response to JKH10, which suggests that the mechanism for the response to JKH10 is not competitively blocked by SR141716 even at supramaximal concentrations (Figure 3D). Pretreatment with pertussis toxin (PT), which blocks the interaction of Gi family proteins with GPCRs, significantly attenuated the JKH10-induced inhibition from 54 ± 2.6% to 110 ± 6.3% of the forskolin-stimulated activity (Figure 3E) but did not affect the JKH1 response (73 ± 11% to 61 ± 7.8%). This finding demonstrates that the mechanism for the inhibition of cAMP accumulation for JKH10 results from activation of a Gi-coupled receptor, whereas the much less efficacious response to JKH1 does not require Gi coupling.

### 2.3. Effects of JKH1 and JKH10 on Extracellular Signal-Regulated Kinase (ERK1/2) Phosphorylation

**Figure 4:**
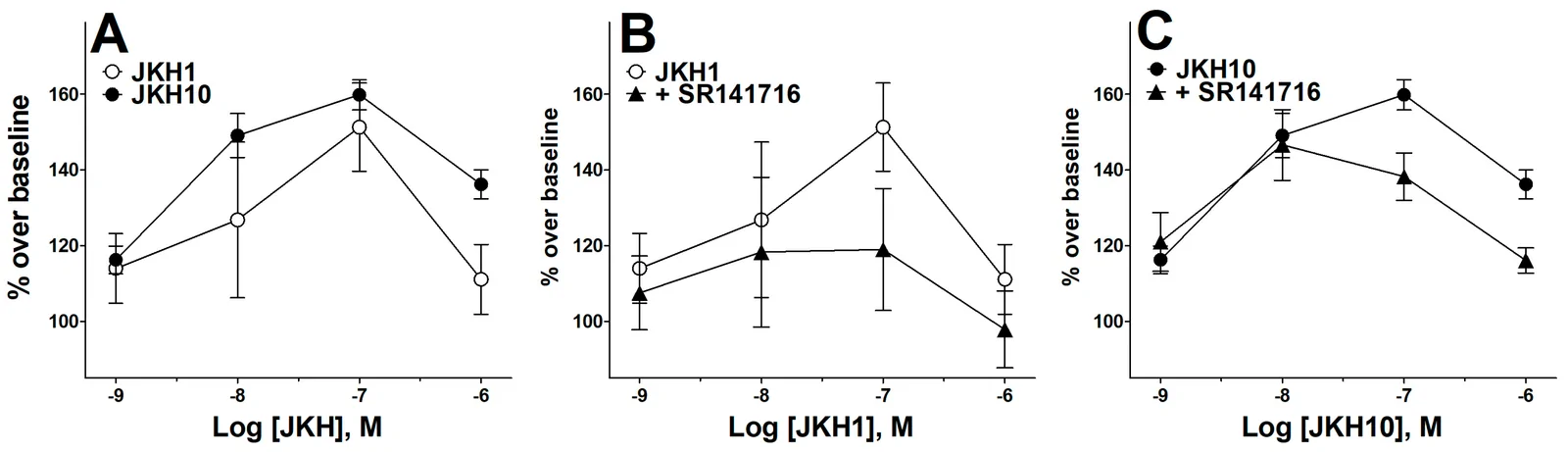
Effects of JKH1 and JKH10 on pERK1/2 accumulation. N18TG2 cells were treated with varying concentrations of JKH1 or JKH10 (**A**). Effects of the CB_1_ antagonist–inverse agonist SR141716 (SR, 1 μM) on JKH1 ((**B**), three independent experiments) or JKH10 ((**C**), five independent experiments) are also shown. Data are presented as the mean ± SEM.

In intact neuronal cells, incubation with JKH1 or JKH10 for 4 min stimulated ERK1/2 phosphorylation. Both analogs stimulated dose-dependently between 1 nM and 100 nM (Figure 4A). These stimulations were reduced by co-incubation with SR141716 (1 µM), but inhibition was not overcome by increasing the concentrations of JKH1 or JKH10 as expected for competitive antagonism (Figure 4B,C). JKH10 appeared to be less sensitive to SR141716, particularly at lower concentrations (Figure 4C). The stimulation decreased at 1 μM JKH1 and JKH10, creating a U-shaped log dose–response curve. This could result from desensitization of the response at higher concentrations and due to the presence of phosphatases within the cell. Alternatively, a signaling pathway that reverses the ERK stimulation may be stimulated by these compounds at higher concentrations. This is not likely to be an “anti-histamine” response because there is no evidence for H1 (or H2) histamine receptors in N18TG2 cells. However, P2 × 7 purinergic receptors are expressed endogenously in N18TG2 cells and can be inhibited by the anti-inflammatory N-alkylpiperazine and diarylmethane, oxatomide (3-[3-(4-benzhydrylpiperazin-1-yl)propyl]-1H-benzimidazol-2-on), resulting in blocking the ATP-driven Ca^2+^ current that may be involved in the activation of mitogen-activated protein kinases [9].

### 2.4. Cannabinoid Effects of JKH1 and JKH10 on Vascular Responses to Noradrenaline in Mouse Mesenteric Arteries

**Figure 5:**
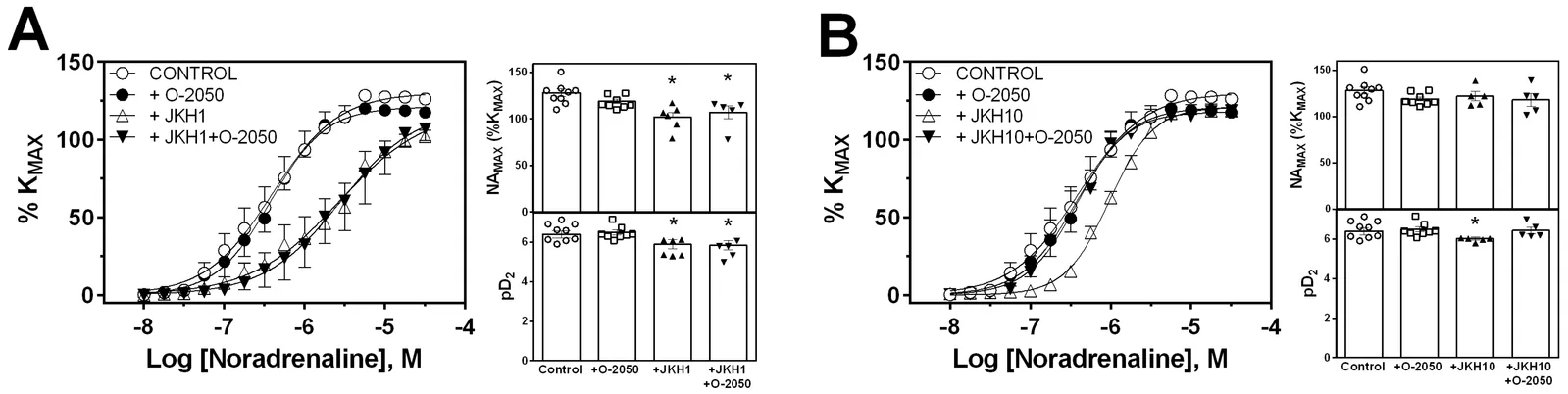
Cannabinoid effects of JKH1 and JKH10 on mesenteric arteries. Mesenteric arteries were exposed to increasing concentrations of noradrenaline (10 nM to 30 μM) in control conditions (n = 9) and in the presence of O-2050 1 μM (n = 8). Incubations with (**A**) JKH1 (1 μM, n = 6) or (**B**) JKH10 (1 μM, n = 5) are indicated as well as co-incubation with O-2050 1 μM and JKH1 (n = 6) and JKH10 (n = 5) were also performed. Data are presented as the mean ± SEM. * *p* < 0.05 vs. Control.

We identified a cannabinoid-dependent response in the mouse mesenteric arteries using Δ^9^-tetrahydrocannabinol (THC) and a full cannabinoid agonist [4]. Consistent with our previous reports [3], both JKH1 and JKH10 attenuate noradrenaline-mediated contraction in mesenteric arteries. JKH1 diminished both maximal noradrenaline response and sensitivity, whereas JKH10 significantly attenuated sensitivity (Figure 5A,B). Preincubation with the CB_1_ orthosteric antagonist-inverse agonist O-2050 (1 μM) had different effects on the actions of JKH1 and JKH10. Whereas O-2050 reversed the effects of JKH10 on noradrenaline sensitivity (Figure 5B), it did not impact the effects of JKH1 on the noradrenaline responses (Figure 5A). These results suggest that in the vasculature JKH1 and JKH10 can attenuate adrenergic contractions, with the effects of JKH10 being inhibited by a CB_1_ antagonist–inverse agonist.

### 2.5. Docking of JKH1 and JKH10 to the CB_1_ Cannabinoid Receptor

**Figure 6:**
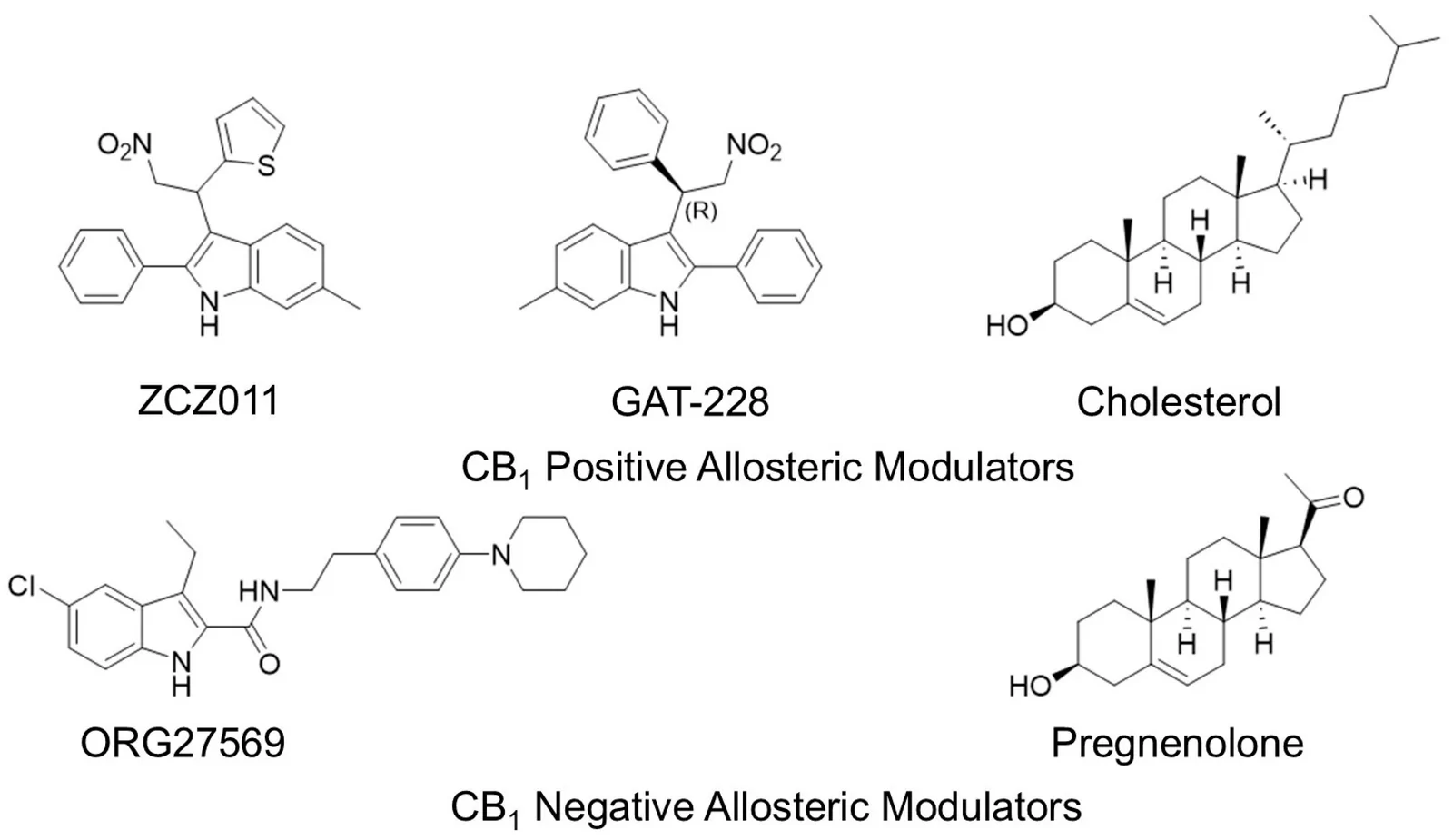
Structures of CB_1_ receptor-positive and -negative allosteric modulators. Strategies to promote medicinal effects of CB_1_ receptor stimulation while reducing untoward side effects include targeting cellular signaling pathways by augmenting or inhibiting ongoing endocannabinoid signaling using positive or negative allosteric modulators, respectively [16,17,18].

**Figure 7:**
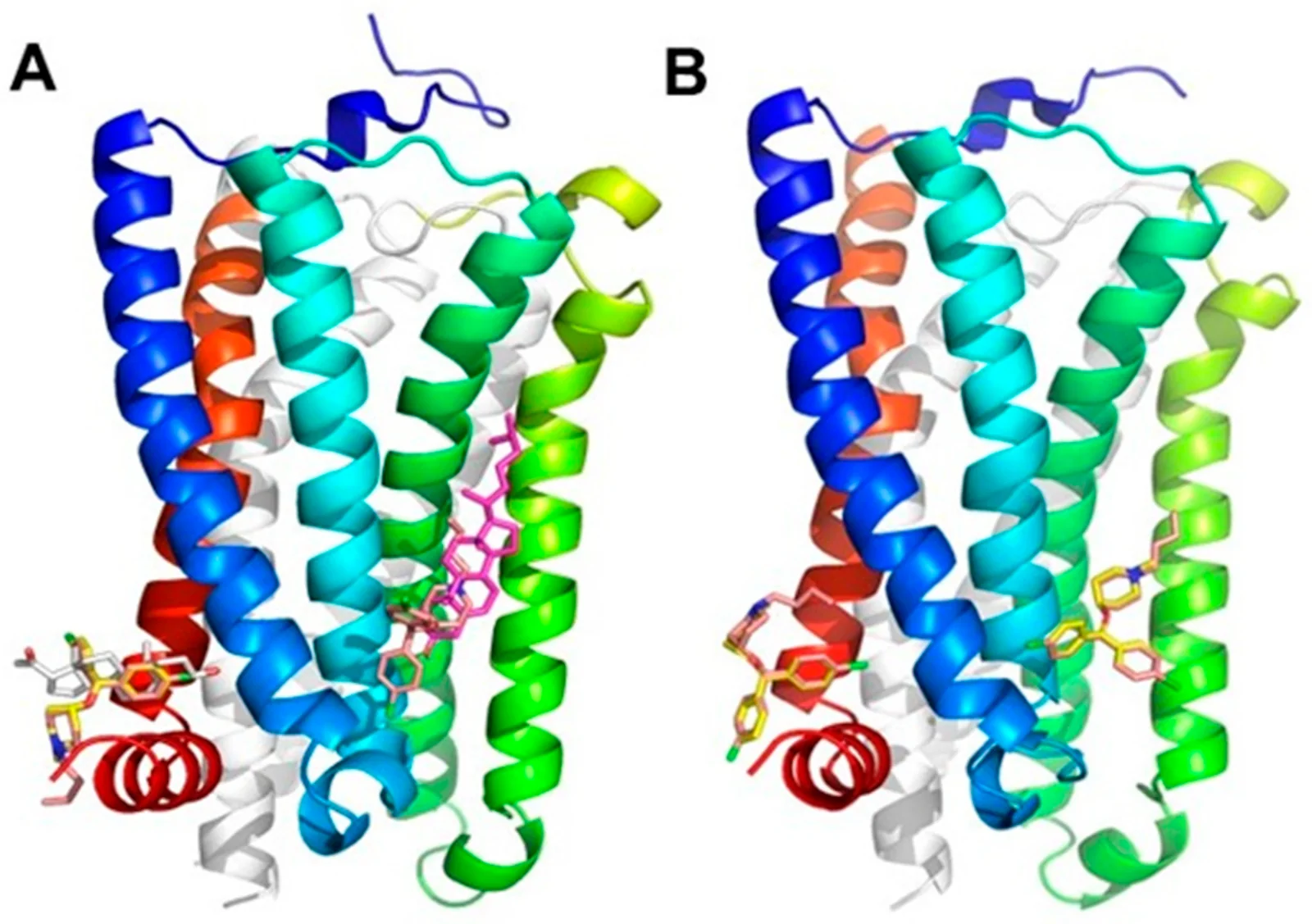
Docking poses of JKH1 and JKH10 on the CB_1_ structure. JKH1 (yellow), JKH10 (salmon), cholesterol (magenta), and pregnenolone (grey) in the PDB structure 7FEE are shown in (**A**), and 6KQI shown in (**B**). CB_1_ receptor helices are colored blue (H1), cyan (H2), dark green (H3), light green (H4), orange (H7) and red (H8).

JKH1 contains a para-Cl atom on one of the phenyl groups and a methyl group bound to the N atom on the piperidine ring. JKH10 contains a para-Cl atom on each phenyl moiety and an n-butyl group bound to the N atom in the piperidine ring (Figure 1). This latter chemical group confers higher lipophilicity to JKH10, exemplified by a log *p* value of 5.931 versus 3.815 for JKH1. This increased lipophilicity may well indicate greater ability of JKH10 to bind within hydrophobic channels or lipid grooves on the lipid-facing surface of the CB_1_ receptor. Among the newer pharmacological tools to study CB_1_ signaling, ZCZ011 and its analog GAT228 are characterized as positive allosteric modulators [10], while ORG27569 is an allosteric modulator that inhibits G-protein signaling but induces downstream ERK phosphorylation, exemplifying the CB_1_ allosteric ligand-biased signaling process [11]. Additionally, pregnenolone [12] and cholesterol [13] have been described as CB_1_ negative and positive allosteric modulators, respectively (Figure 6). We applied a docking algorithm to discern the interaction of the two compounds, with the CB_1_ receptor in crystal structures bound to CP55940 in the orthosteric site and ZCZ011 in a “positive allosteric site” (7FEE) [14], or CP55940 in the orthosteric site and ORG27569 in a “negative allosteric site” (6KQI) [15] (Figure 6 and Figure 7).

Molecular interactions of diphenylpyraline derivatives with the CB_1_ receptor were identified from the results of a cluster analysis using the 7FEE crystal structure (Figure 7 and Table 1). The results predict that JKH1 binds primarily in the pregnenolone binding site, while JKH10 binds to both the pregnenolone and cholesterol binding sites. Neither compound appeared at the site where the positive allosteric modulator ZCZ011 was in the crystal structure at helices 2–3–4 facing the outer leaflet of the plasma membrane [19]. JKH1 docked with <5% probability at the orthosteric binding pocket for CP55940, calculated from the number of observed poses in the docking simulations.

**Figure 8:**
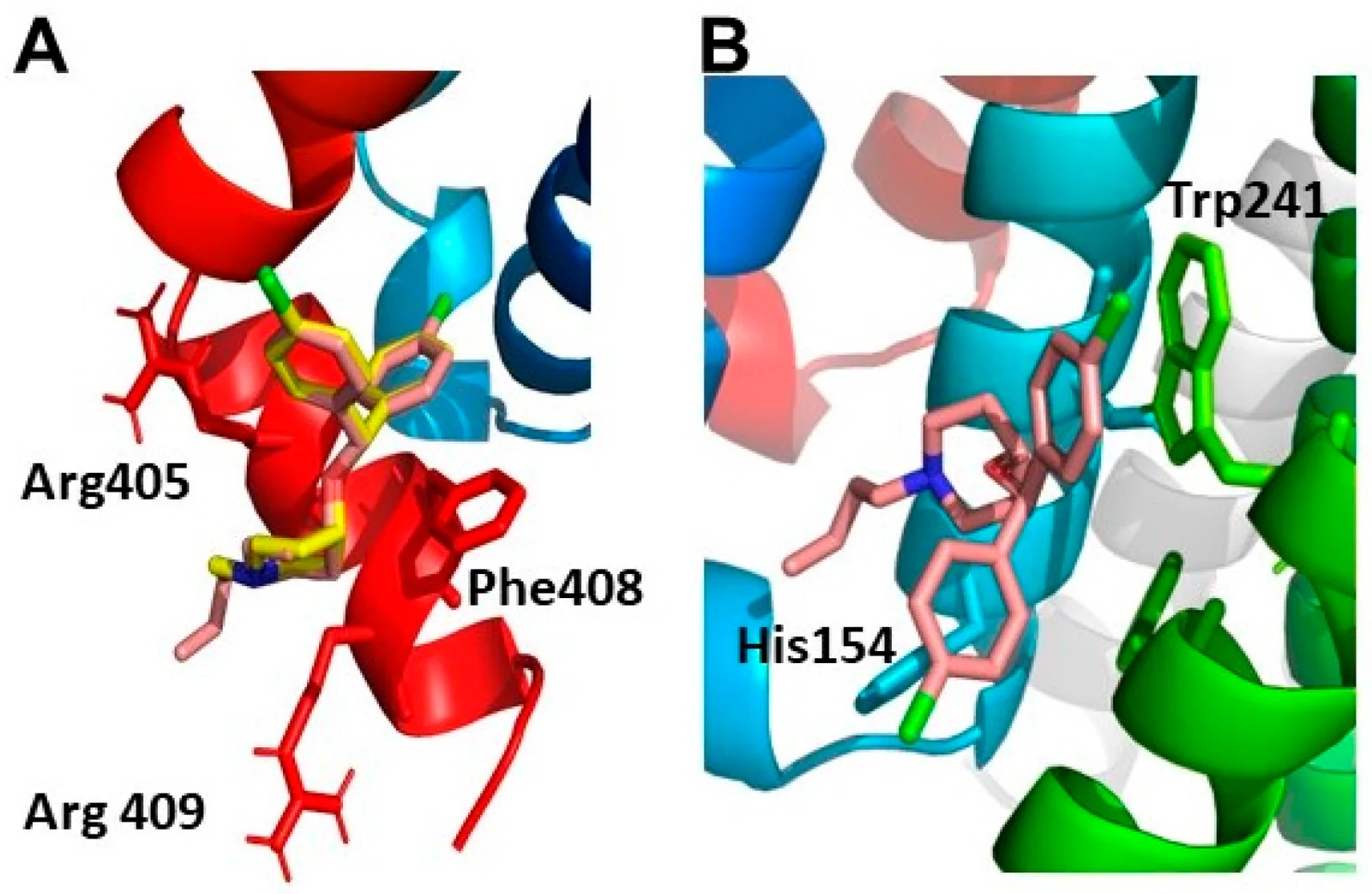
Molecular interactions of JKH1 and JKH10 with the CB_1_ receptor. Residues identified in the PDB structure 7FEE are predicted to be important for the binding of the JKH compounds in the pregnenolone site (**A**) and the cholesterol site (**B**). Refer to Figure 7 for helixes colors.

**Figure 9:**
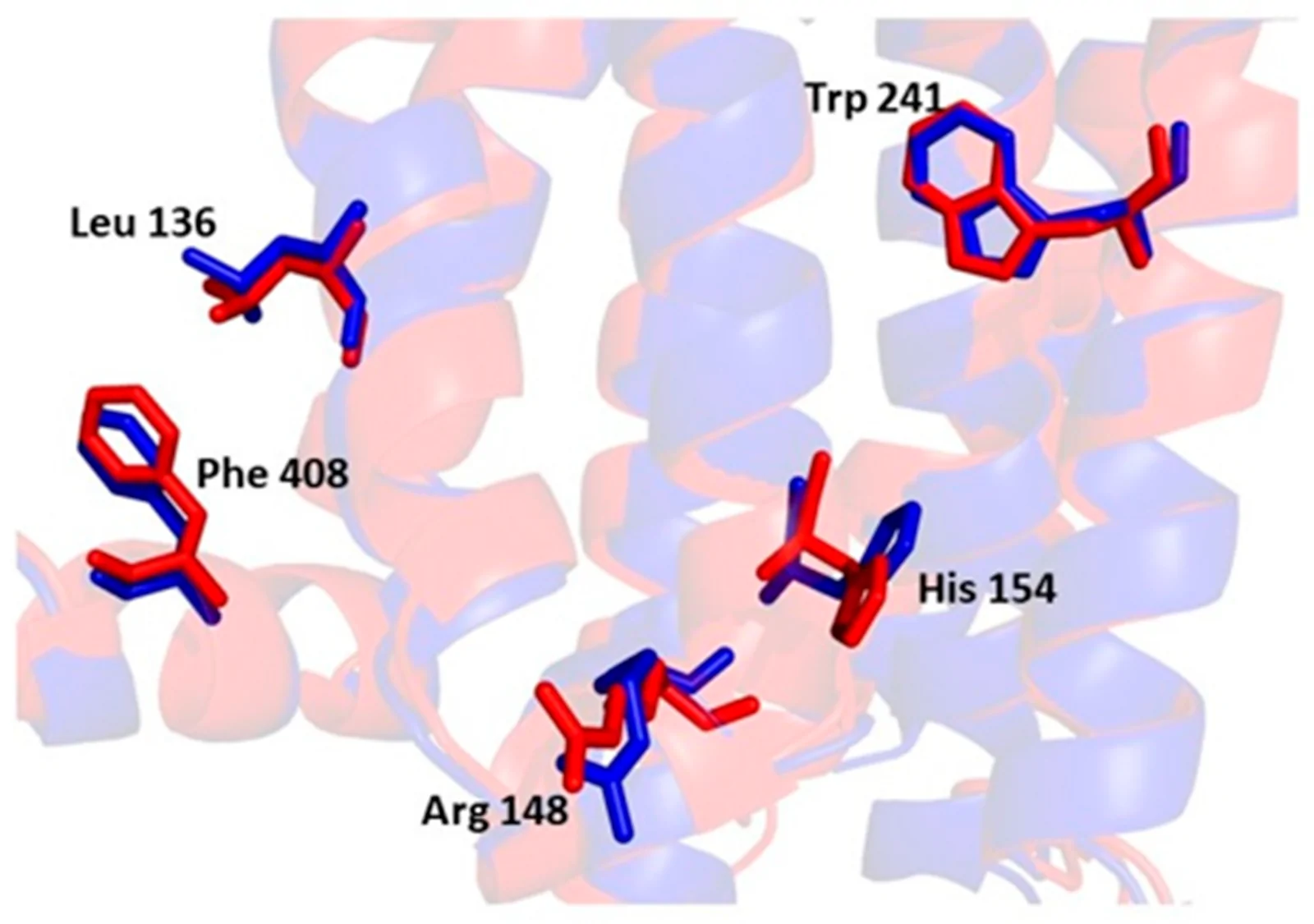
Residues on the CB_1_ receptor interacting with JKH1 and JKH10. Alignment of the 7FEE (red) and 6KQI (blue) CB_1_ receptor structures results in an RMSD of 0.781 angstroms. Residues predicted to interact with the JKH compounds are shown.

The binding pockets identified in the docking studies were consistent with previously described sites [15] (Figure 8 and Figure 9). The pregnenolone site was defined by Val140, Leu136, Glu133, Ile395, Ala398, Leu404 Arg405 and Phe408. The cholesterol-binding site was bounded by Leu138, Leu143, Arg148, Trp241, His154, Ser158, Val161 and Leu165. The phenyl rings of JKH1 and JKH10 are situated to form an aromatic stacking interaction with Phe408, and the butyl moiety is buried in a hydrophobic region near Leu136 in the pregnenolone site. The chlorine of JKH10 is equidistant from Arg148 and His154 in the cholesterol binding site, and the other phenyl ring is stacked with Trp241.

## 3. Discussion

In this investigation, we have explored JKH1 and JKH10, two members of a series of diphenylpyraline analogs that exhibited different capabilities in stimulating cellular signaling via G protein activation, Gi-mediated inhibition of cAMP accumulation, rapid-phase ERK phosphorylation and relaxation of mesenteric artery constriction. A pharmacological analysis of these diphenylpyraline analogs shows that JKH10 exhibits signaling stimulation much like the orthosteric agonist WIN55212-2 but differs in showing non-competitive, but not competitive, antagonism to the CB_1_ receptor-selective orthosteric antagonist–inverse agonist SR141716. Although JKH1 stimulates ERK phosphorylation, this analog shows poor efficacy in stimulating G protein activation or inhibiting Gi-mediated inhibition of cAMP accumulation.

We infer that JKH10 can stabilize the population of receptors in their active state because it promotes stimulation of G protein activation and Gi-mediated inhibition of cAMP accumulation and stimulates ERK1/2 phosphorylation in a similar manner to a CB_1_ receptor agonist. We determined that the receptor is the CB_1_ cannabinoid receptor because the JKH10 stimulation is sensitive to a selective CB_1_R antagonist capable of promoting the inactive state of the CB1 receptor.

In the present study, *in silico* docking studies were not able to locate docking of JKH10 to the orthosteric binding site (that locus of the receptor that binds the endogenous ligand and its analogs (CP55940, WIN55212-2) within two models of the CB_1_ receptor derived from crystal structures, suggesting that JKH10 is not acting at the orthosteric site. This is supported by the observation that the antagonism of the JKH10 response was not competitive. Rather, docking was localized to two sites that were identified as allosteric sites (loci that bind ligands but not at the orthosteric site for endogenous ligand and its analogs) for modifying the activity of the CB_1_ receptor. One site is reported to bind pregnenolone and the other to bind cholesterol. The simplest interpretation consistent with the results is that JKH10 behaves as a stimulator to increase the population of active CB_1_ cannabinoid receptors and that this activation occurs through either a pregnenolone- or a cholesterol-binding site or both to promote an active state of the CB_1_ receptor.

Like WIN55212-2, JKH10 dose-dependently stimulated the binding of a GTP analog to Gi proteins in neuronal cells that endogenously express CB_1_ cannabinoid receptors, and efficaciously inhibited FSK-stimulated cAMP accumulation and stimulated ERK1/2 phosphorylation. Unlike WIN55212-2, JKH10-mediated inhibition of cAMP accumulation and the stimulation of ERK1/2 phosphorylation were non-competitively, but not competitively, inhibited by SR141716, i.e., were subject to the inverse agonist but not competitive effects of this CB_1_-selective inhibitor. These properties are like those of ZCZ011, which acts as an allosteric agonist in both G protein- and β-arrestin-driven pathways. ZCZ011 behaves as a positive allosteric modulator in only the β-arrestin-driven responses [20].

A full agonist can be competitively inhibited by SR141716 because the antagonist and agonist bind at the same orthosteric binding site (see [21]). However, SR141716 produced a non-competitive GTPγS binding response for JKH10, consistent with SR141716′s preclusion of the progression of the activation process by JKH10. Non-competitive antagonism can be identified because the binding sites for JKH10 and for SR141716 differ, so there can be no competition for the orthosteric binding site occupied by SR141716. It is not clear what micro-conformational changes are utilized by JKH10 to achieve activation. However, published reports indicate that SR141716 can stabilize the CB_1_ receptor in its inactive state by mechanism(s) that include aromatic stacking interactions with F3.36/W6.48, which consequently stabilizes transmembrane helix 6 in its inactive conformation [22,23,24]. Additionally, the carbonyl of SR141716 acts as a hydrogen bond acceptor and stabilizes the D6.58 (366)-K3.28(192) salt bridge in the CB_1_ receptor inactive state [22]. These mechanisms could preclude the formation of an active state in both orthosteric and allosteric agonism, making it possible to hypothesize that the mechanism of activation by JKH10 is similar to that of an orthosteric agonist.

JKH1 inhibited G protein activation with low potency and efficacy and was poorly effective at Gi-mediated inhibition of cAMP accumulation. Regarding cAMP accumulation, JKH1 was not influenced by SR141716 or pertussis toxin treatment and evoked neither inhibition nor augmentation of the maximal WIN55212-2 response. Even so, the JKH1-mediated ERK1/2 phosphorylation was non-competitively inhibited by SR141716 across all concentrations. These findings suggest a bias in its stimulation of ERK phosphorylation versus Gαi-mediated cAMP production.

Because inhibition of adenylyl cyclase is a direct result of CB_1_-mediated activation and the release of Gαi proteins, the observed differences in exerting this signaling could be based upon differences in the ligands’ mechanisms to promote the CB_1_ receptor’s engagement, activation, or subsequent release of the Gαi protein, or the ability of Gαi to interact with adenylyl cyclase. These findings could also indicate that the diphenylpyraline derivatives are not subject to the competition of SR141716 at the orthosteric site that binds cannabinoid or aminoalkylindole agonist ligands.

The regulation of ERK1/2 signaling pathways is a complex involvement of GPCRs and receptor tyrosine kinases and/or cytosolic tyrosine kinases. In the N18TG2 neuronal model, the CB_1_ receptor-mediated early-phase ERK1/2 phosphorylation is initiated via Gβγ and intracellular tyrosine kinases [25]. Thus, a signaling bias for JKH1 to promote CB_1_-mediated ERK1/2 phosphorylation could occur by promoting Gβγ scaffolding, even in the absence of an efficacious Gαi-mediated reduction of cAMP. CryoEM structures of the CB_1_ receptor–Gi heterotrimer fail to distinguish the intracellular protein interactions important for signaling [19,26]. However, *in silico* modeling has predicted a CB_1_ receptor C-terminal–Gβγ interface that could serve a functional role in directing signaling pathways [27]. Alternatively, the ERK1/2 phosphorylation could have been initiated by scaffolding to β-arrestin via a mechanism that requires the helix 7 NPxxY motif near the elbow with helix 8 [28].

It is often difficult to translate molecular signaling at the cellular level to complex physiological functions that are regulated by many diverse autocrine and endocrine factors. In our study, we have investigated the cannabinoid-mediated vasorelaxation of noradrenaline-constricted mouse mesenteric resistance arteries [3,4]. In this preparation, JKH10 shifted the log dose–response curve for constriction to the right with no change in the slope factor, and the JKH10 response was totally reversed by the CB_1_ receptor-selective antagonist O-2050. This can be interpreted to result from a CB_1_ receptor-mediated signaling mechanism. In contrast, JKH1 shifted the log dose–response relationship to the right and reduced the slope factor, but neither property was altered by O-2050. The decreased slope factor is consistent with more than a single mechanism in the regulation of the process, including more than a single influence on CB_1_ receptor conformation or signaling. It is certainly possible that these diphenylpyralines promote vasorelaxation via a non-CB_1_ receptor mechanism. More recently, we showed that JKH1 and JKH10 have the potential to inhibit Ca^2+^-mediated vascular contractions [29].

We identified two primary sites of interaction of the JKH analogs with the CB_1_ receptor: (1) both JKH1 and JKH10 docked to a site that was identified by pregnenolone binding, and (2) JKH10 docked to a site that recognizes cholesterol. Pregnenolone was characterized as a negative allosteric modulator because it antagonized the Δ^9^-THC-mediated phosphorylation of ERK1/2 in HEK293 or CHO cells transiently expressing CB_1_ receptors [12]. However, this signaling was “biased” because there was no antagonism of the Δ^9^-THC-mediated decrease in cAMP accumulation in CB_1_-expressing CHO cells [12,16]. JKH1 appears to be a biased agonist toward ERK1/2 phosphorylation rather than cAMP regulation in N18TG2 cells endogenously expressing CB_1_ receptors.

The pregnenolone-binding site was identified computationally in a rhodopsin-homology model of the CB_1_ receptor by a Forced-Biased Metropolis Monte Carlo-simulated annealing program to be at the helix 1–helix 7 region near the elbow forming helix 8 [12]. In that model, Glu133 in helix 1 at the elbow region of the CB_1_ receptor was predicted to hydrogen bond with the ketone of pregnenolone, and this prediction was supported by observations that Δ^9^-THC-stimulated respiration in CB_1_-expressing HEK293 was abrogated by an E133G mutation [12]. CB_1_ Leu136 and helix 8 Phe408 were predicted to form hydrophobic interactions, and helix 8 Arg405 was predicted to form hydrogen-bonding interactions with the hydroxyl in pregnenolone [12]. These researchers proposed that the hydrogen bonding with polar amino acids at both ends of pregnenolone could bind in a way that precludes helical movement in the receptor, resulting in its “negative modulation”. In our rigid model of the CB_1_ receptor in conformations selected by both orthosteric agonist and allosteric modulators, the hydroxyl is oriented outward toward Arg405, but the ketone is buried deeper in the pocket than Glu133. JKH1 and JKH10 would not be predicted to confer rigidity in this region of the receptor, which may facilitate JKH1-biased agonist behavior.

JKH10 binds to a cholesterol consensus motif (CCM) located at helices 2–3–4 facing the inner leaflet of the plasma membrane, a common domain to many class A GPCRs [30,31]. In the crystal structure with agonist-bound (5XRA), cholesterol is found in that site [32]. Trp241 could interact with cholesterol [32], as it does in our model for JKH10. From this site, cholesterol can influence ligand binding to the orthosteric site of the CB_1_ receptor [31,33]. Cholesterol has been proposed as an endogenous CB_1_-positive allosteric modulator [19]. This CCM site is a location for both positive and negative modulation of the CB_1_ receptor activation state. Computational studies predicted that the (R) enantiomer GAT228, which is an analog of ZCZ011 (also enantiomeric), binds to this CCM site [34]. In that model, interactions with Trp241 and His154 were predicted to promote an active receptor conformation, consistent with the behavior of GAT-228 as an allosteric agonist [34]. This CCM site was also identified as the binding site for the negative allosteric modulator ORG27569 in the crystal structure with CP55940 bound at the orthosteric site (6KQI), as well as in computational studies [15,34]. This location was confirmed by site-directed mutagenesis and probe-binding studies [31]. Trp241 could form a hydrogen bond with ORG27569 to stabilize an inactive receptor conformation [15]. It has also been predicted that one mechanism of action for cannabidiol as a CB_1_ negative allosteric modulator is due to cannabidiol binding to this CCM site [35]. When ORG27569 binds to this allosteric site, it displaces the cholesterol and behaves as a “negative allosteric modulator” [15,36]. JKH10 has a different shape and interacts with different amino acid residues within that same binding pocket but must facilitate the active conformation of the receptor because JKH10 behaves as an allosteric agonist.

Several diphenylpyraline analogs were powerful reuptake inhibitors at the dopamine transporter compared to cocaine [2]. Previous studies of our group also showed the ability of these analogs to inhibit the serotonin transporter (Supplemental Material Table S1). Structure–activity relationship studies revealed that compounds with lipophilic N-substituents exhibited a decrease in IC_50_ values relative to DPP. Halogenation in the para position on the diphenyl methoxy moiety gave rise to this same trend. Both aliphatic and halogen meta-substituents led to an increase in the IC_50_ relative to DPP and para-substituted analogs. We now know that the dopamine transporter contains cholesterol bound to a CCM site required for functionality [37,38,39]. It will be necessary to reassess the series of diphenylpyraline analogs for their mechanism via this cholesterol binding site.

Amphipathic and lipophilic molecules are being discovered to gain access to GPCRs via lateral diffusion within the plasma membrane and bind to the transmembrane helices along the outside of the helical bundle or enter the transmembrane bundle partially or fully from their entry point at the junction of the plasma membrane and the protein [40]. The modification of GPCRs by relatively conserved, intra-membrane allosteric sites is a contemporary target for drug design for medicines that can influence the activation state in the absence of an orthosteric agonist (allosteric agonists or antagonists) or modify the activity of the endogenous ligand (positive or negative allosteric modulators) [41,42]. Our identification of JKH10, which behaves as an allosteric agonist, can add to the growing list of compounds that can help researchers understand the micro-conformational changes leading to GPCR activation but may also identify novel opportunities for pharmacotherapeutics beyond the orthosteric ligands.

## 4. Materials and Methods

### 4.1. Cell Culture, Membrane Preparation, [^35^S]GTPγS Binding to G Proteins

N18TG2 neuroblastoma cells were maintained and harvested as previously described [43]. N18TG2 membranes were prepared, and [^35^S]GTPγS binding was performed as described [44]. Cell membranes (5 μg protein) were incubated for 1 h at 30 °C with 500 pM [^35^S]GTPγS (PerkinElmer, Billerica, MA, USA), 10 μM GDP and CB_1_ ligands in 20 mM Na-Hepes, pH 7.4, 100 mM NaCl, 5 mM MgCl_2_, and 1 mM dithiothreitol in a 96-well plate. The reaction was terminated by filtration through a UniFilter plate using a Top Count cell harvester (Model D961962; PerkinElmer, Billerica, MA, USA), with extensive washing with ice-cold TME buffer, and scintillation cocktail was added to the UniFilter plate. Radioactivity was quantitated in a TopCount micro-plate scintillation counter (Model C384V00; PerkinElmer, Billerica, MA, USA). The nonspecific binding was measured in the presence of 10 µM unlabeled GTPγS and subtracted from total binding.

### 4.2. cAMP Assay

cAMP assays were performed on N18TG2 cells as described [44]. Briefly, cells were grown to 90% confluence in 24-well plates. The cells were washed with a Na-Hepes-buffered saline solution containing bovine serum albumin before being incubated with phosphodiesterase inhibitors (3-isobutyl-1-methylxanthine and rolipram) and ligands for 15 min at 37 °C. Following this, forskolin (FSK, Tocris Bioscience, Minneapolis, MN, USA) was added for an additional 4 min before stopping the assay. cAMP was measured as previously described [43].

### 4.3. ERK1/2 Phosphorylation Immunocytochemistry Assay

PhosphoERK1/2 (pERK1/2) protein levels were quantified using a 96-well format “In-cell-Western” immunocytochemistry technique as described previously [44]. Briefly, N18TG2 cells were seeded at a density of 30,000 cells per well, grown until 90% confluent, serum-starved for 16 h, and pretreated with the diacylglycerol lipase inhibitor tetrahydrolipstatin (1 μM) to attenuate the production of 2-arachidonoylglycerol, for 2 h prior to incubation. Cells were treated with vehicle or ligands at indicated concentrations at 37 °C for 4 min. After treatments, media were removed, and cells were immediately fixed and permeabilized with 100 µL ice-cold 100% methanol for 10 min with gentle rocking. After rinsing twice with phosphate-buffered saline, cells were blocked in LI-COR blocking buffer (LI-COR Biosciences^®^, Lincoln, NE, USA) and Na vanadate for 2 h at room temperature before staining with pERK rabbit polyclonal antibody (1:100) (4370S; Cell Signaling, Danvers, MA, USA) for 18 h at 4 °C. After rinsing twice with tris-buffered saline-0.1% Tween-20 (TBST), cells were incubated with secondary antibody donkey anti-rabbit (1:500) and DRAQ5 (1:1000) (Invitrogen, Waltham, MA, USA) for 75 min at room temperature. The plate was then rinsed with TBST, and immunofluorescence was imaged using the LI-COR Odyssey (LI-COR Biosciences^®^, Lincoln, NE, USA). Basal and stimulated expression of pERK was calculated as the ratio of fluorescence of pERK1/2 to DRAQ5.

### 4.4. Animals

Male C57BL/6 mice (3 months old, n = 9) were sacrificed under anesthesia by cervical dislocation. The mesenteric vasculature was isolated and kept in Krebs–Henseleit Buffer (KHB) containing: 118 mM NaCl, 4.47 mM KCl, 25 mM NaHCO_3_, 1.2 mM KH_2_PO_4_, 1.2 mM MgSO_4_, 2.5 mM CaCl_2_·2H_2_O, and 5.5 mM glucose, on ice until use. Animal procedures were reviewed and approved by the Campbell University Institutional Animal Care and Use Committee.

### 4.5. Vascular Reactivity to Noradrenaline

Vascular reactivity was measured in isolated mouse mesenteric arteries using wire myography. Arterial segments with a maximum length of 2 mm were mounted on a multi- myograph (Model 620M, DMT-USA Inc, Ann Arbor, MI, USA) using two 40 μm stainless steel wires as described previously [45]. The myograph chamber was maintained at 37 °C, filled with 5 mL KHB and oxygenated with a gas mixture containing 95% O_2_/5% CO_2_. After washing and resting for 30 min, each arterial segment was normalized using consecutive steps of stretching, and the resulting wall tension-internal circumference curve (DMT Normalization Module; ADInstruments, Colorado Springs, CO, USA) was used to calculate optimal diameters (OD). Arteries were normalized at a level of stretch producing 90% of the internal circumference a vessel has when experiencing a transmural pressure of 100 mm Hg (0.9·L_100_). The average OD of the mesenteric arteries used in this study, calculated as 0.9·L_100_/π, was 194 ± 5 μm. After resting for an additional 30 min, arteries were exposed to a cumulative dose–response curve of noradrenaline (10 nM–30 μM) in fourth-log steps. Each dose was administered sequentially, following stabilization of the prior response. Parallel assays involved pre-incubating arteries for 20 min with JKH1 or JKH10 (1 µM), or with the analogs together with O-2050 (1 µM), before assessing noradrenaline-induced concentration-response effects. Data were acquired using Powerlab (ADInstruments, Colorado Springs, CO, USA) and the LabChart Pro v8 software (ADInstruments, Colorado Springs, CO, USA).

### 4.6. Molecular Modeling

Ligand structures were generated and protonated using Open Babel 3.1.1 at a simulated pH of 7.3, followed by 3D coordinate generation and energy minimization using the MMFF94 force field. The CB_1_ structures were obtained from the Protein Data Bank (PDB) for 7FEE (the CB_1_ receptor structure that was crystallized bound to CP55940 and the positive allosteric modulator ZCZ011) and 6KQI (the CB_1_ receptor structure that was crystallized bound to CP55940 and the negative allosteric modulator ORG27569). Both CB_1_ structures were prepared with AutoDock Tools 1.5.6 by removing all waters and heteroatoms, adding polar hydrogens, and assigning Kollman charges. Co-crystallized ligands were removed from the PDB structures and included in the ligand library to validate the docking method. Rigid molecular docking was performed using AutoDock Vina 1.1.2. The search space (grid box) was centered on coordinates: *x* = 24.475 *y* = 3.869 *z* = 42.264, with dimensions of *x* = 40 *y* = 62 *z* = 40 Å for 6KQI, and coordinates: *x* = −0.462 *y* = 18.367 *z* = 1.932, with dimensions of *x* = 60 *y* = 40 *z* = 68 Å for 7FEE, using an exhaustiveness value of 8. A maximum of 20 binding modes were generated for each ligand for cluster analysis, with an energy difference cutoff of 4.0 kcal/mol from the best-scoring pose [46]. Protein–ligand interactions and docking conformations were visualized and analyzed using PyMOL 3.1.8 [47]. Rigid docking was considered appropriate for this binding-site search because the objective was not to rank closely related ligands within a single, well-defined binding pocket, but rather to identify plausible receptor surface regions that could accommodate JKH1 and JKH10. In this context, the analysis was used as a site-localization approach rather than a quantitative comparison of binding affinities. The CB1 structures used for docking already represent experimentally determined receptor conformations stabilized by orthosteric and allosteric ligands, thereby providing relevant static conformational states for evaluating whether the diphenylpyraline derivatives preferentially occupy known pregnenolone- or cholesterol-associated regions. Because JKH1 and JKH10 were not docked for direct potency comparison within the same binding site, receptor flexibility was less critical than it would be for discriminating against small energetic differences among competing ligands in a common pocket. Thus, rigid docking was adequate for identifying candidate allosteric interaction sites, while the docking scores should be interpreted qualitatively and in conjunction with the pharmacological evidence rather than as precise estimates of binding free energy.

### 4.7. Data Analysis

All data were analyzed using Microsoft Excel and GraphPad Prism v8 software. Ligand-stimulated log concentration–response curves were determined as the percent change relative to FSK-stimulated values expressed as 100% (cAMP accumulation) or relative to basal expressed as 100% for vehicle-treated cells (ERK1/2 phosphorylation). Statistical analysis was performed using one-way ANOVA followed by Dunnett’s post-hoc test. For the vascular dose–response curves, maximal response to noradrenaline (NA_MAX_) and sensitivity (pD_2_ = −Log[EC_50_]) were determined by fitting the responses of individual arteries to the logistic curve Y  =  bottom + (top − bottom)/(1  +  10(LogEC_50_ − X) × Hill Slope), where X is the logarithm of the concentration and Y is the response. Statistical analysis was performed using one-way ANOVA followed by Dunn’s multiple comparisons test. Data are expressed as mean ± SEM; a *p* < 0.05 was accepted as an indication of statistical significance.

## 5. Conclusions

Our studies demonstrated the ability of two diphenylpyraline derivatives JKH1 and JKH10 to stimulate cellular signaling responses that can be non-competitively inhibited by CB_1_ receptor-selective inverse agonists SR141716 and O-2050. *In silico* analysis demonstrated that neither docked at the orthosteric agonist binding site in crystal structures reported for the CB_1_ receptor. In contrast, JKH1 and JKH10 were predicted to dock at locations of the CB_1_ receptor reported for binding to pregnenolone or a cholesterol motif. The unique docking to protein surface sites within the plasma membrane and functional activity of these structurally different compounds have identified a novel class of CB_1_ receptor allosteric stimulators or biased allosteric stimulators that may have the ability to behave as regulators of other integral membrane proteins via a comparable lipid crevice or cholesterol binding site.

By investigating the diverse biology of these DPP analogs, we serendipitously have discovered that the commonality between the CB_1_ receptor and perhaps other GPCRs and the monoamine transporters is the cholesterol consensus motif and/or the pregnenolone-binding site. This insight is an important step toward investigating the role that cholesterol might play in regulating these proteins, but also in other integral membrane proteins that can respond to their lipid environment with functional changes. The present investigation has identified the JKH compounds as potential tools to investigate important regulatory mechanisms such as the role of high cholesterol found in lipid rafts or the functioning of annular lipids in the regulation of these proteins.

## Figures and Tables

**Table 1:** Docking scores (Kcal/mol) for the interactions of JKH ligands with the pregnenolone and cholesterol site in the CB_1_ receptor.

CB_1_ Receptor Sites (PDB 7FEE)	Calculated Docking Scores (Kcal/mol)			
	JKH1	JKH10	Pregnenolone	Cholesterol
Pregnenolone site	−6.7	−6.5	−9.0	n/a
Cholesterol site	−6.5	−7.1	n/a	−8.8

## Data Availability

Docking results can be made available through Figshare at tereeves@wakehealth.edu.

## Supplementary Materials

The following supporting information can be downloaded at https://www.mdpi.com/article/10.3390/molecules31203699/s1: Table S1: DPP and JKH diphenylpyraline derivatives characteristics.
