# Supplementary material for: Diphenylpyraline Derivatives Are Allosteric Agonists of the CB1 Cannabinoid Receptor
Source: Molecules. 2026 Oct 15;31(20):3699. doi: 10.3390/molecules31203699 (PMC13707696; doi:10.3390/molecules31203699)
Supplement: Supplementary file 1 [file molecules-31-03699-s001.zip › molecules-4431950-supplementary.pdf]

Supplementary Material

Diphenylpyraline Derivatives Are Allosteric Agonists of the CB_1 Cannabinoid Receptor

Khalil Eldeeb [1,2,3], Jill K. Harp [2,4,5,6], Ryan W. Fitzgerald [4], Sandra Leone-Kabler [2], Tony E. Reeves [7], Sara R. Jones [2], Steve R. Childers [2], Allyn C. Howlett [2], Victor M. Pulgar [4,8,9,*]

1 Jerry M. Wallace School of Osteopathic Medicine, Campbell University, Lillington, NC 27506, USA; keldeeb@methodist.edu
2 Department of Physiology & Pharmacology (Translational Neuroscience), Wake Forest University School of Medicine, Winston-Salem, NC 27157, USA; sandra.leonekabler@advocatehealth.org (S.L.-K.); sara.jones@wfusm.edu (S.R.J.); srchilders928@gmail.com (S.R.C.); allyn.howlett@advocatehealth.org (A.C.H.)
3 Faculty of Medicine, Al-Azhar University, New Damietta 34518, Egypt
4 Biomedical Research Infrastructure Center, Winston-Salem State University, Winston-Salem, NC 27101, USA; harpj@wssu.edu (J.K.H.); fitzgeraldrw@wssu.edu (R.W.F.)
5 Department of Biological Sciences, Winston-Salem State University, Winston-Salem, NC 27101, USA
6 Department of Chemistry, Winston-Salem State University, Winston-Salem, NC 27101, USA
7 Wake Forest Institute for Regenerative Medicine, Wake Forest University School of Medicine, Winston-Salem, NC 27157, USA; tony.reeves@advocatehealth.org
8 Department of Obstetrics and Gynecology, Wake Forest University School of Medicine, Winston-Salem, NC 27157, USA
9 Department of Pharmaceutical & Clinical Sciences, Campbell University, Lillington, NC 27506, USA
* Correspondence: pulgar@campbell.edu; Tel.: +1-910-8931701

This file contains a supporting table including structure and formula of diphenylpyraline (DPP) and the JKH series of diphenylpyraline derivatives. The Table also includes data generated by our group [1,2,3] showing inhibition of the dopamine (DAT, IC_{50}) and serotonin (SERT, K_i) transporters, sensitivity (pD_2) for noradrenaline responses in mesenteric arteries, as well as cAMP accumulation and [^{35}S]GTPγS binding values for selected diphenylpyraline derivatives. Calculated logP (cLogP) is also indicated.

Table S1. DPP and JKH diphenylpyraline derivatives characteristics.

Structure	Name & Formula	DAT IC_{50} (nM)	SERT K_i (nM)	Motion[1] (%)	NA pD_2[2]	cAMP[3] (% inh)	GTP[4] (nM)	cLogP
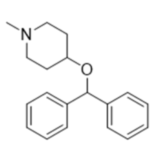	**DPP** $C_{19}H_{23}NO$	420±9.0	13587±1920	311.0	5.38±0.04	5.5	-	3.206
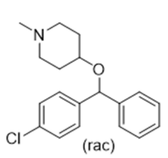	**JKH1** $C_{19}H_{22}ClNO$	155±10.0	2426±130	159.4	5.08±0.08	31.25	2	3.919

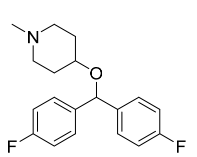	**JKH2** $C_{19}H_{21}F_2NO$	22.1±5.7	7518±1690	887.8	5.10±0.12	18	-	3.492
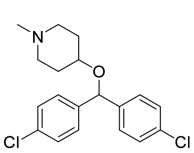	**JKH3** $C_{19}H_{21}Cl_2NO$	44.0±10.9	>14,000	150.2	-	-	-	4.632
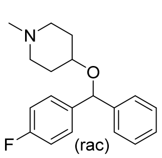	**JKH4** $C_{19}H_{22}FNO$	277±10.0	3083±330	126.5	-	-	-	3.349
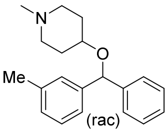	**JKH5** $C_{20}H_{25}NO$	293±10.0	5534±1720	88.8	-	-	-	3.705
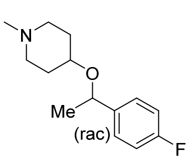	**JKH8** $C_{14}H_{20}FNO$	264±9.0	>14,000	28.9	-	-	-	2.140
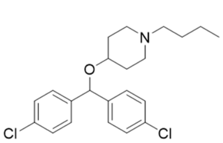	**JKH10** $C_{22}H_{27}Cl_2NO$	12.5±7.5	396±57	98.7	4.26±0.07	60.25	0.466	6.219
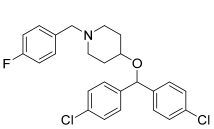	**JKH11** $C_{25}H_{24}Cl_2FNO$	32.0±1.4	>15,000	182.0	-	51.25	-	5.079
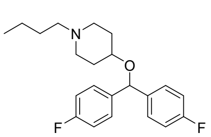	**JKH12** $C_{21}H_{25}F_2NO$	61.6±7.2	>15,000	140.5	-	-	-	4.330
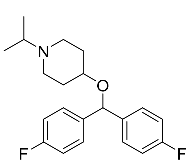	**JKH13** $C_{21}H_{25}F_2NO$	61.6±7.2	>15,000	140.5	4.48±0.15	-	-	4.330
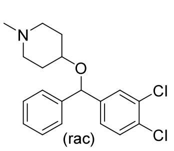	**JKH14** $C_{19}H_{21}Cl_2NO$	5353±160	>14,000	91.7	-	-	-	4.512

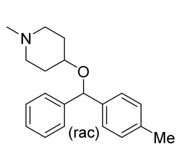	**JKH15**<br>$C_{20}H_{25}NO$	6000±150	>15,000	99.3	-	-	-	3.705
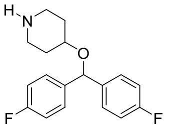	**JKH16**<br>$C_{18}H_{19}F_2NO$	17±2.0	>15,000	206.7	5.17±0.07	38.5	-	3.046
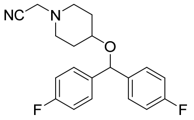	**JKH17**<br>$C_{20}H_{20}F_2N_2O$	251±12.0	>14,000	8.5	-	57.75	-	2.904

Structure and formulas of DPP and JKH diphenylpyraline derivatives, inhibition of the dopamine (DAT) and serotonin transporter (SERT) are also shown. [1]Locomotor measurements expressed as % of basal after a dose of 17.8 μmol/kg [1,2]. [2]NA pD_2, sensitivity to noradrenaline, all values significantly lower than control (5.95±0.03) [3]. [3]cAMP accumulation measured as % inhibition of forskolin-dependent cAMP levels. [4]GTP: EC_{50} for $[^{35}S]GTP\gamma S$ binding to G proteins. The partition coefficient derived from the chemical structure (cLogP) using ChemDraw® (Revvity Signals Software, Inc., Waltham, MA, USA) is also indicated. -: not determined.
